# Comparison Effect of Midazolam Alone and Midazolam Combined with Ketamine in Bone Marrow Aspiration Pain in Children

**Published:** 2015-07-20

**Authors:** H Mahmoudi Nesheli

**Affiliations:** **Non-Communicable Pediatric Diseases Research Center, Babol University of Medical Sciences, Babol, Iran.**

**Keywords:** Bone Marrow Aspiration, Lumbar Puncture, Ketamine, Midazolam

## Abstract

**Background:**

This study aimed to compare sedative and analgesic effects of oral Midazolam and Ketamine on Bone Marrow aspiration (BMA) and Lumbar Puncture (LP).

**Material and Methods:**

This study was a randomized clinical trial and was performed in Amirkola Hospital in north of Iran, Babol during 2011 and 2012 .The study population consisted of 40 patients who underwent the first time of diagnostic BMA for any reason, patients were divided randomly in two groups: Oral Midazolam and combined Oral Midazolam and Ketamine. Each group consisted of patients with age of 3-7 years and over 7 years .Two methods of pain status and soothing were evaluated through CAMFORT scale checklist based on MAGNUSON National Institutes of Health Medical Center. Statistical analyses were done by Spss v.19.

**Results:**

In our study, 17(42.5%) and 23(57.5%) were female and male, respectively. 28(70%) patients were aged between 3 and 7 years and 13(30%) older than 7 years. The obtained findings revealed that the difference between Midazolam sedation and combination of Midazolam and Ketamine sedation was significant (P= 0.00). The sedation in older patients was more than young patients in combination of Midazolam and Ketamine group. (P= 0.22).

**Conclusion:**

These findings showed that Ketamine and Midazolam combination had more efficacy than Midazolam alone for decreasing pain and sedation.

## Introduction

International Association Of Pain defend pain as a discomfort sensory from emotional experience associated with actual or strong damage (1). For children, the pain is not the just an uncomfortable feeling but also it can cause confusion because the child can not foresee the pain and often do not understand it (2). Children's ability to understand the pain varies with their age. Younger children show more pain than older children (1). Due to the excessive importance of pain and it´s control, Pain Society of America declared that pain is the fifth vital sign and decade 2001 - 2010 as the Decade of Pain Control called Although, it is recommended that pain management is the key element in enhancing quality of child^’^s cares, but a large number of children still experience unbearable levels of pain (3). On the other hand, painful procedures are uncomfortable for parents and health workers. Parents often are anxious seeing their ailing children and this procedure is a problem for health workers too (4) . This procedure is would also cause hurt in the relationship between health professionals and children (5).With improving the treatment of cancers in children, the pain associated with the treatment is more prominence that pain associated with cancer (6). Pain can cause physiological changes such as increased heart and breathing rates, sweating, redness of the skin, decreased oxygen saturation, dilated pupils, restlessness, and hypertension (7). If it is not controlled, it lead to more complications such as life and different body systems, and agitation, anorexia, incontinence, restlessness, insomnia, fear at night (8-10). While, Psychological side effects as learning and memory impairment and mental illness may occur in the future (11). Invasive procedures such as biopsy, BMA and lumbar puncture (LP) are as an integral part of the diagnostic and therapeutic procedures in children with hematologic malignancies. These procedures are painful and bearing BMA and LP often harder than disease itself (12).Therefore, creation of sedation and analgesia are increasingly expanding for diagnostic and therapeutic measures in the fields of hematology and oncology (13). For this purpose, in addition to psychological support programs, pharmaceutical methods are used for sedation and analgesia of these children (14-16). Given the short duration of these actions, it is necessary to use medications or combination of medications that relieve the pain and anxiety of patients (17-18). A number of studies also recommends use of Midazolam before performing an invasive procedure to reduce anxiety and fear in children (19). This study aimed to compare sedative and analgesic effects, the level cooperation of the patient and parents ´satisfaction of oral Midazolam with a combination of Ketamine and oral Midazolam in patients who underwent BMA conduction. Thalassemia major is an inherited disease most prevalent in the region called thalassemia belt (1). Iran is one of the countries located on the belt with an average thalassemia gene prevalence rate of 4% (2). Upon the establishment of Iran Thalassemia Association in 1989, the need was felt to make measures for the prevention of thalassemic newborns in Iran (2). As a result, the first country-wide thalassemia prevention program was formulated in 1995 and started to be implemented across Iran in 1997 (3). The first study about the outcomes of the program was conducted by Samavat et al. which showed significant success in decreasing the rate of thallasemic newborns (4). Subsequent studies showed the exaggerated success reported in the first study due to the delay in the registration process of newborn thalassemic patients in Iran Health System which had not been considered by the researchers (5)^. ^The second effort to evaluate the thalassemia prevention program in Iran was made by Abolghasemi et al. who considered two different phases in their study: the first phase during which the screening of marriage candidates was the only strategy to reduce the rate of thalassemia and the second phase in which the abortion in special medical cases received religious approval which made the use of prenatal diagnosis (PND (possible in prevention of thalassemia. They concluded that the former phase was not that successful and the latter phase on the contrary was success (3). However, no statistics have been reported in their study to show their definition of success. Hadipour et al. in another study evaluated the success rate of the thalassemia prevention program during 2001-2006 period indicating the downward trend in the number of thalassemia new births as a success, though with heterogeneity in a few provinces, where the program was faced with serious challenges (5). In yet another study conducted by Hadipour et al., the emphasis has been placed again on the heterogeneity of the program in different provinces, though they evaluated the program to be successful (6). Miri et al. also studied about the success rate of the program in comparison with the neighboring Muslim countries and a few European countries like Greece and Cyprus; their findings showed the program to be successful as a model for the prevention of blood-borne diseases in developing Muslim countries (2). But there is not yet any consensus on the success of the program in Iran and many other researchers like Ghotbi et al. hold the belief that the program has many obstacles in its way to fulfill its goals (7). In the present review, given the importance of thalassemia prevention in the countries being located on the thalassemia belt, and considering the importance of the Iranian prevention program as a reliable model for developing Muslim countries, we have reviewed the success of the program considering its various aspects including the change it has made in the attitude of the society, the decrease in the number of thalassemia new births, and the effectiveness of the network of PND laboratories in Iran. 

## Material and Methods

This randomized trial study was done in Department of Oncology and Hematology Center in Amirkola Children’s Hospital in north of Iran, Babol, during 2011- 2012. The Study population consisted of 40 patients who underwent the first time of diagnostic BMA in Oncology Ward for any reason. They were divided randomly in two groups: Oral Midazolam and combined Oral Midazolam and Ketamine. Each group consisted of patients with age of 3- 7 years and over 7 years. Classification criteria of ASA consist of five classes: Class I: no systemic disease, Class II: mild systemic disease, Class III: Severe systemic disease, Class IV: Systemic disease Which is life-threatening and Class V: life is not possible with or without surgery. Exclusion criteria were: Patients with life-threatening systemic diseases, chronic heart and lung disease, patients who suffered from respiratory infection, patients treated with anti epileptic drugs, patients aged less than 3 years, and finally patients who had allergy to benzodiazepines. The first group was given 0.2 mg / kg oral Midazolam (with Exir brand Company) 30 minutes prior to aspiration and second group was given 5 mg/kg of oral Ketamine (with Rotex brand) accompanied with 0.2 mg/kg oral Midazolam in sweet solution half an hour before the procedure. Before the procedure, complete explanation was given to the parents and consent was received. Fasting conditions were not taken into consideration for our patients. Pain and relax status were evaluated for children during a checklists based on CAMFORT Scale related to the National Institutes of Health, that include 8 items and each item was evaluated with 5 scores, include: Alertness ,Calmness ,Crying,Physical Movement ,Facial Tension, Muscle Tone, Blood Pressure,Respiratory Rate and Heart Rate . Before doing the procedure, child's heart rate and blood pressure were measured. Then, within 5 minutes after the procedure, blood pressure and heart rate were measured. Side efeccts of Ketamine and Midazolam such as hallocination and Apneawere recorded. All items of check list were recorded by nurse without the knowledge of prescribed drugs. Check list was prepared according to the scale and scoring, sedative effects and pain decrease of these two methods. Findings were analyzed by using SPSS Software V.19 and statistical T-Test. P-value of less than 0.05 was considered significant.

## Results

In this study 17(42.5%) were female and 23(57.5%) were male, and 28(70%) of patients were aged between 3 – 7 years and 13(30%) were older than 7 years. The obtained findings revealed that the difference between Midazolam sedation and combination of Midazolam and Ketamine sedation was significant (P= 0.00). This means that patients who received the combination therapy in comparison to patients who received Midazolam alone experienced more soothing level. The difference of patients´ cooperation and satisfaction of parents in tow groups, were significant (P=0.00). It means that, patients´ cooperation and parents´ satisfaction in combination therapy group and satisfaction with their parents in this group were more than Midazolam group alone. The results of the analysis of data showed that the effect of age in patients´ sedation in M-K group was significant (P=0.014) It means that in combination group, level of sedation increased with age (P= 0.22).In our study, there was no case of Apnea and other breathing difficulties, double vision. Hallucinations that happened in few patients were quickly away. The results of differences in two methods in terms of sedation in both genders showed no significant differences (P>0.05). Table1 presents comparison of different variables in two groups of patients.

**Table I:** Descriptive and analytical statistical findings in two groups of patients

	**Degree of freedom**	**T score**	**Standard deviation**	**Mean**	**GGroups**	**P**
**Sedation level of ** **patients**	38	4.78	4.5 6.7	33.25 24.60	Combined group Midazolam only	0.000
**Collaboration rate of patients**	38	4.58	12.75 3.05	12.75 8.95	Combined group Midazolam only	0.000
**Satisfaction of ** **patients´ parents**	38	4.17	0.74 1.23	4.15 2.80	Combined group Midazolam only	0.000
**Sedation level of** **Patients based on patients´ age**	18	2.73	6.9 2.29	22 29.42	Combined group 3-7 >7	0.014
	18	2.73	4.61 3.78	32.53 35.40	Midazolam only 3-7 >7	0.22
**Sedation level of ** **Patients based on patients´ gender**	18	0.41	5.77 7.61	23.88 25.18	Combined group Girl boy	0.68
	18	0.49	4.35 4.74	33.87 32.83	Midazolam only Girl boy	0.62

## Discussion

Many factors can affect on the life children with malignancy and their families (20). Pain and it´s control are very important. Pain and fear of bodily injury caused by it, have high prevalence among individuals, especially among children. There were significant differences among levels of sedation, patient cooperation rate for BMA and parental satisfaction based on American Society of Anesthesiologists (ASA) between two methods of oral Midazolam alone and oral Midazolam and Ketamine combined. That is similar to other studies nevertheless; the type of procedure applied for patients was varied compared to current study (21-24). The main focus of the current study was only use of two drugs that are taken orally, which was very easy method and requires no operating room and without spending a lot of time and without fasting of children. But in many studies, the drugs were used intravenous, intramuscular or rectal forms, that in addition to being bothersome in doing of job, it was associated with parental dissatisfaction for doing procedure and increased complications (22-23). In this study, there was no case of Apnea and other breathing difficulties, double vision. Hallucinations happened in few patients. Parental satisfaction was very high in our study. In this study, there was significant difference in the amount of sedation in only Midazolam therapy in the age group 3 to 7 years and older than 7 years, That means, we can study the efficacy of Midazolam in the lower age group with further studies. If this hypothesis will be confirmed by more registration numbers of patients, it can be avoided with this method from side effects of Ketamine. However, most studies showed that Midazolam is less effective for pain relief, on the other hand, the studies not include different ages(22, 25-26). But our result does not apply at in ages over than 7 years, and it is necessary to entail combination treatment of Midazolam and Ketamine. There was is no significant difference between two genders for Midazolam, and combination of Midazolam and Ketamine, this subject did not investigated in other studies. Unlike Banerjee, who advises Midazolam alone to reduce cancer child's fear, Our study rejected the use of Midazolam alone before a painful procedure because Midazolam alone does not provide adequate analgesia (21). We suggest combination of oral Midazolam-Ketamine approach to reduce pain and the child's BMA fear. Our study had some problems and limitations such as a limited first cases who underwent BMA, the restriction of the inclusion and exclusion criteria, limitations due to the necessity of the procedure by a person, problems associated with parental justification, problems associated with double blind study of non-completion questionnaire for nursing and nurse prescribing.

## Conclusions

Midazolam and Ketamine are effective way to reduce the pain and fear children and increasing parental consent in the performance of invasive procedures such as the BMA. This method was very easy and uncomplicated, being efficient and it could solve the problems of children and their families in performing BMA and intrathecal treatment. We suggest to all hospitals that treat children with cancer to give Midazolam-Ketamine combination orally, half an hour before BMA and IT procedures.
